# Digital imaging of crack evolution in granite containing unparallel flaws under fatigue loading

**DOI:** 10.1038/s41598-026-47334-8

**Published:** 2026-04-13

**Authors:** Vahid Kordloo, Kamran Goshtasbi, Hamid Reza Nejati, Alireza Bokaei, Mehrdad Mahmoodi

**Affiliations:** 1 Department of Rock Mechanics, School of Engineering, Tarbiat Modares University, Tehran, Iran; 2 Department of Mechanical Engineering, Tarbiat Modares University, Tehran, Iran; 3 Department of Mining and Metallurgy Engineering, Amirkabir University of Technology, Tehran, Iran

**Keywords:** Jointed rock mass, Crack propagation, Digital image correlation, Cyclic loading, Non-persistent joints, Failure mechanism, Engineering, Materials science, Solid Earth sciences

## Abstract

Failure in jointed rock masses presents a major challenge in tunneling, mining, and rock slope engineering, typically occurring along surfaces formed by interacting cracks at pre-existing flaws. Understanding how cracks initiate, propagate from these flaws, and eventually coalesce can contribute to more reliable and safer rock structure design. This study investigates the influence of the unconfined compressive monotonic and cyclic loading conditions and varied unparallel flaw geometries on the crack propagation process and mechanical properties of granite. Five distinct flaw geometries were analyzed, each containing two pre-existing flaws. The upper flaw (Flaw number ①) had a fixed inclination angle of 45°, while the lower flaw (Flaw number ②) was oriented at 0°, 45°, 90°, 135°, and 180°, both relative to the horizontal axis. Based on the orientation of Flaw number ②, the specimens were labeled S0, S45, S90, S135, and S180. These configurations ranged from non-overlapping geometries (S0, S45, and S90) to overlapping ones (S135 and S180). The analysis was conducted under monotonic and then cyclic loading conditions with stress amplitude set at 75%, 80%, and 85% of the peak uniaxial compressive strength (${\sigma }_{c})$ obtained from monotonic tests for each geometry. Crack development was captured using Digital Image Correlation (DIC) via GOM Correlate software, enabling high-resolution monitoring of surface strain and displacement fields at the pixel level. The results reveal that flaw geometry significantly influences the crack coalescence path and final failure pattern. A transition from non-overlapping to overlapping flaw configurations shifted the crack coalescence from indirect to direct trajectories. Cyclic loading, in some cases, causes changes in crack propagation paths at lower amplitudes, as well as the formation of new cracks, referred to as cyclic cracks, unique to this type of loading, leading to distinct overall failure patterns compared to other loading types. While the loading type (monotonic vs. cyclic) had minimal effect on the mechanism of crack initiation, it notably altered the sequence of crack growth and propagation mechanism. Mechanical properties also varied with both geometry and loading conditions. Under monotonic loading, overlapping geometries exhibited higher strength and a greater crack initiation stress ratio (CI/CP, defined as the ratio of crack initiation stress to the peak compressive strength of the specimen) compared to non-overlapping ones. Specifically, specimens S90 and S45 demonstrated the highest values of ${\sigma }_{c}$ (161 MPa) and CI/CP at 51%, respectively. In contrast, S180 showed the lowest values, approximately 124 MPa in strength and 17% in CI/CP. Finally, flaw geometry was found to govern both fatigue life and its sensitivity to loading amplitude. As the loading amplitude increased from 75 to 80% and 85%, fatigue life decreased across S0, S45, S90, and S135. Notably, S180 exhibited non-linear behavior, with the fatigue life dropping from 425 to 257 cycles at 75–80%, then increasing slightly to 310 cycles at 85%.

## Introduction

Crack propagation in jointed rock masses presents significant challenges to the stability of structures in geotechnical engineering, including tunneling^1^, mining^2^, and slope stability^3^. The mechanical behavior of these rock structures is fundamentally governed by the interaction and propagation of pre-existing flaws under different loading conditions, where stress redistribution around excavations intensifies local stress concentrations near discontinuities^4,5^. This makes flaw geometry and the specific loading regime the primary factors controlling crack initiation, propagation, coalescence, and ultimately failure^6^.

For decades, foundational research successfully identified several crack types initiating from pre-existing flaws, often focusing on specimens containing a single flaw or parallel flaws under monotonic compression^7–13^. These studies established a qualitative framework for wing cracks and secondary cracks, demonstrating that parameters such as flaw inclination and ligament length strongly influence initiation thresholds and coalescence patterns^14^. However, natural rock masses rarely contain isolated or perfectly parallel flaws, and the proximity of multiple unparallel discontinuities leads to much more complex stress redistribution and crack interaction than single-flaw models can explain^15,16^.

Natural discontinuity systems are generally composed of unparallel fissures which produce stress fields significantly different from parallel systems^17^. When flaws are unparallel, the interaction between tips and the surrounding matrix is asymmetrical, causing local stress fields and crack trajectories to evolve in ways not typically observed in parallel configurations^17,18^. For instance, Wei et al.^17^ reported that wing cracks frequently deviate from their typical paths and “bend” when approaching adjacent unparallel flaws, suggesting that stress shielding and interaction are more pronounced in these geometries. Haeri et al.^18^ further demonstrated through numerical and experimental modeling that the Stress Intensity Factors (SIFs) and initiation angles at each tip are highly sensitive to the relative orientation of the second flaw, meaning a small change in flaw angle can completely shift the coalescence path. Similarly, Lee and Jeon^19^ investigated unparallel fissures in PMMA and granite, observing that while tensile cracks dominate the early stages, the subsequent shear interactions and coalescence trajectories are uniquely governed by the geometric arrangement and the material’s grain-level behavior^19^. Despite these findings, the underlying mechanisms governing the chronological order of interaction in multiple unparallel flaw systems remain insufficiently understood.

Furthermore, the majority of prior studies concentrated on static or monotonic loading conditions^9,13,20^. This approach fails to account for cyclic loading (fatigue), which is critical for rock structures subjected to repeated stresses^21^. Previous fatigue research has explored various parameters, though often using simplified joint arrangements^21^. For instance, Li et al.^22^ utilized gypsum specimens with parallel intermittent joints to show that increasing the loading frequency from 0.2 to 21 Hz slows down damage evolution. Additionally, Xue et al.^6^ employed semi-circular granite specimens to find that tiered constant amplitude (TCA) loading accelerates irreversible damage more than variable amplitude (VA) paths. Regarding multi-flawed systems, Ko et al.^23^ investigated two parallel or overlapping flaws in prismatic gypsum specimens using a constant frequency of 0.5 Hz and a triangular waveform. By varying the maximum stress (80%, 85%, and 90% of dynamic strength), they observed that higher flaw inclination angles and lower maximum applied stresses resulted in a longer fatigue life^23^. While Ko et al.^23^ identified unique horizontal and coplanar “fatigue cracks” that appeared only after a specific number of cycles, they concluded that the overall crack growth sequence and coalescence patterns remained almost identical to monotonic loading for parallel geometries.

Crucially, no study has yet explored how varying cyclic loading parameters, particularly stress amplitude, influences the chronological sequence, initiation, propagation mechanisms, and coalescence behavior in specimens containing multiple unparallel flaws. There is a complete lack of evidence regarding how the direction of propagation and the formation of unique cyclic fractures are dictated by cyclic loading parameter changes in these geometrically complex configurations, nor has their specific fatigue life sensitivity been quantified.

Another important limitation of many previous investigations lies in the observation technique^13^. Conventional methods, such as visual inspection, are often subjective and may fail to capture full-field quantitative data on displacement and strain, especially during early-stage damage accumulation^13,24^. To overcome these limitations, Digital Image Correlation (DIC) has emerged as a precise tool for measuring full-field surface deformation^25^. DIC allows for the identification of strain localization zones that precede visible cracking and provides an objective basis for analyzing crack initiation^26–28^. More recently, DIC-based relative displacement analysis has been utilized to objectively distinguish between tensile (Mode I), shear (Mode II), and mixed-mode initiation mechanisms by analyzing displacement jumps at the pixel level^24^.

The present research aims to fill these gaps by systematically investigating the crack growth behavior in prismatic granite specimens containing varied unparallel flaw configurations under both monotonic and cyclic regimes. The novelty of this study lies in utilizing high-resolution DIC to perform a direct quantitative comparison between the two loading types, specifically focusing on how changes in cyclic stress amplitude dictate the chronological fracture growth sequence and the initiation/propagation mechanisms which have never been examined in unparallel systems. By identifying the specific impact of amplitude on the development of unique cyclic cracks and the resulting coalescence trajectories, this work seeks to provide a comprehensive framework for predicting the fatigue performance of jointed rock structures under complex dynamic conditions.

## Methodology

In this study, granite specimens containing pre-existing flaws were subjected to both uniaxial monotonic and cyclic loading to examine and compare their crack growth behavior and overall failure patterns. The full-field surface displacements and strain evolutions were analyzed using the 2D-DIC technique to provide detailed insight into fracture mechanisms under different loading conditions.

### Experimental setup

**Fig. 1:**
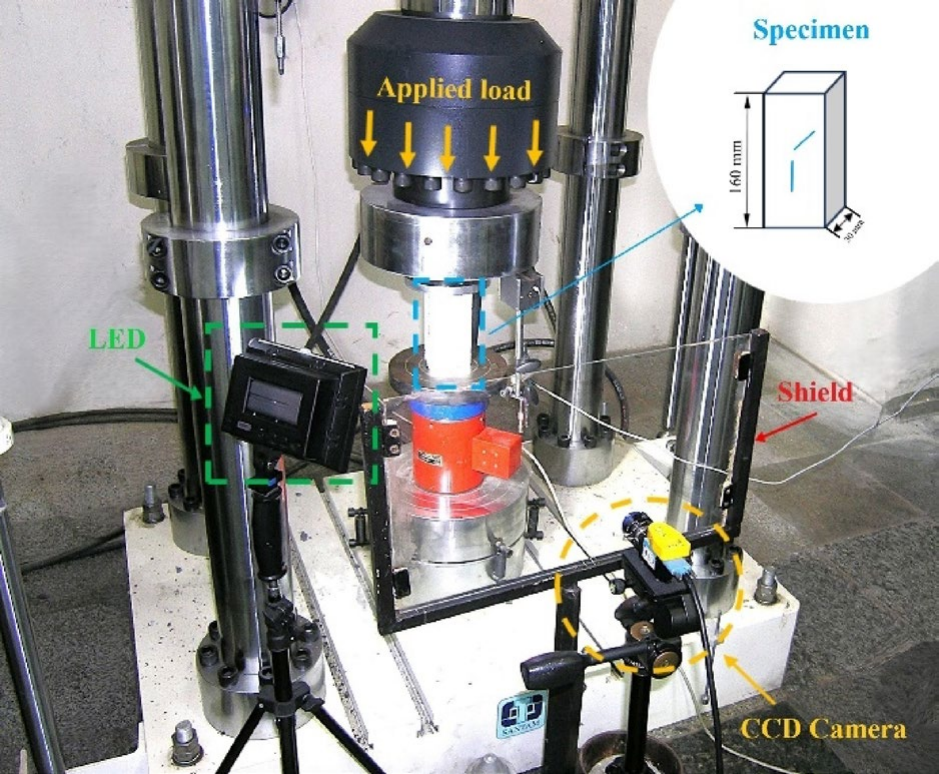
Set up used for test, Loading system, and CCD Camera.

All loading tests were conducted using a SAF-2000 servo-hydraulic testing machine with a 200-ton capacity, capable of applying both monotonic and cyclic loads with high precision. The loading process was fully computer-controlled, allowing for accurate force and displacement measurements. To capture the surface deformation of the specimens, a high-resolution CMOS digital camera (2448 × 2048 pixels, 5 MP) was positioned in front of the specimens. Images were recorded at a frame rate of 1 fps for monotonic loading and 2 fps for cyclic loading. A plexiglass shield and LED lighting system were installed to minimize distortion and ensure consistent lighting (Fig. 1).

**Fig. 2:**
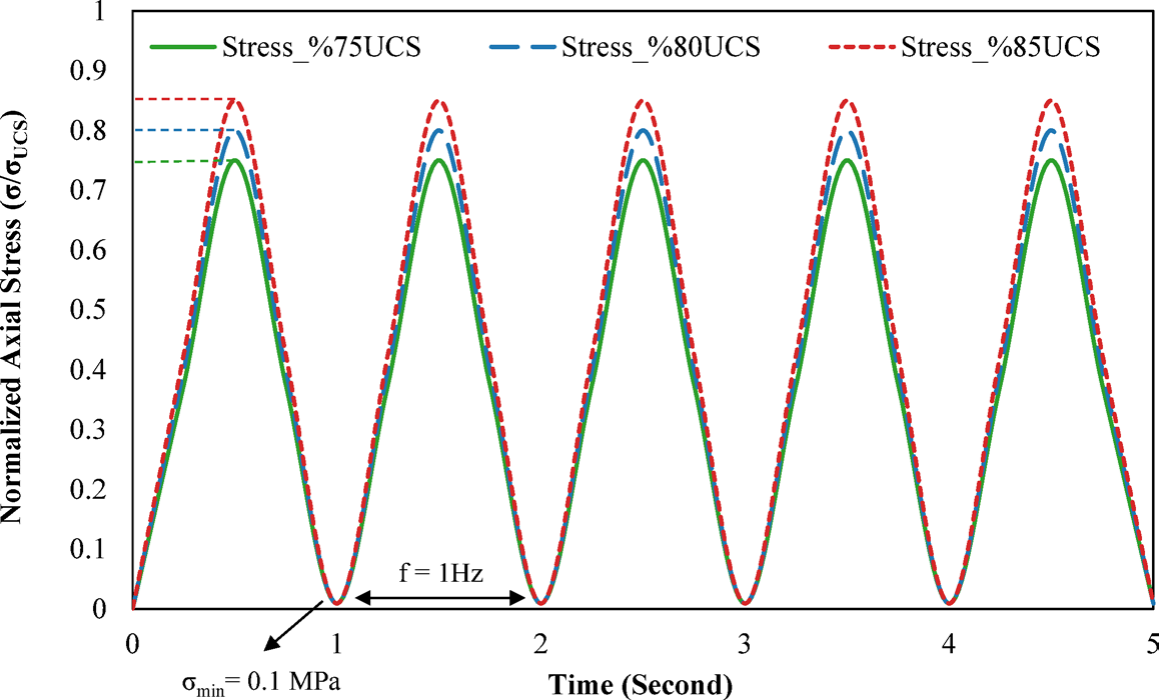
Schematic illustration of the applied loading paths under cyclic loading conditions.

During monotonic loading, a constant strain rate of 0.002 mm/s was applied until specimen failure. Two synchronized data loggers continuously recorded stress–strain data throughout the tests. Cyclic loading parameters were determined based on preliminary uniaxial compression tests. The cyclic loading was performed using a sinusoidal waveform coupled with a 1 Hz frequency to simulate seismic conditions. A minimum axial stress (${\upsigma }_{\mathrm{min}}$) of 0.1 MPa was maintained in all tests to preserve contact between the specimen and loading plates. The maximum stress (${\upsigma }_{\mathrm{max}}$) was set at 75%, 80%, and 85% of the uniaxial compressive strength obtained from monotonic tests. A schematic representation of the cyclic loading paths used in this study is presented in Fig. 2 to improve the clarity of the applied loading protocols.

All parameters in cyclic loading tests were kept constant, except for ${\upsigma }_{\mathrm{max}}$, which was varied to study its influence on crack propagation. The DIC analysis was carried out using GOM Correlate software to extract full-field strain distributions and monitor crack evolution.

### Specimen preparation

Granite blocks were sourced from Natanz, Iran. Specimens were cut and shaped to standardized dimensions (160 mm in height, 80 mm in width, and 30 mm in thickness). The height-to-width ratio was kept at 2 to ensure central stress concentration and stable fracture development. The basic physical and mechanical properties of the tested granite are summarized in Table 1. The symbols used in the table represent density (ρ), P-wave velocity (Vp), uniaxial compressive strength (UCS), Brazilian tensile strength (BTS), Young’s modulus (E), and Poisson’s ratio (ν).

**Table 1:** Basic physical and mechanical properties of Natanz granite.

ρ (kg/m3)	Vp(m/s)	UCS (MPa)	BTS (MPa)	E (GPa)	ν
2650	5200	160	11	38	0.23

**Fig. 3:**
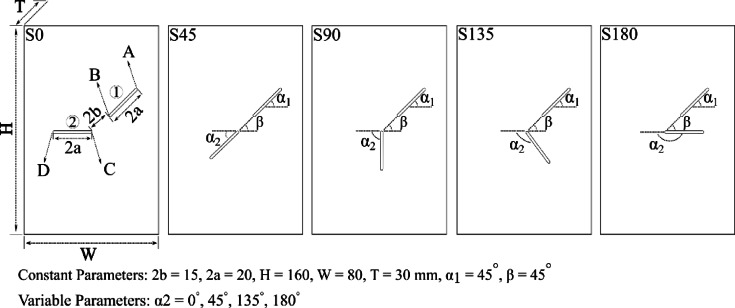
The overall geometry of the specimens and the created Flaws.

To ensure a 2D crack propagation condition, the thickness was limited to 30 mm, as suggested by Yang et al.^29^. Each specimen contained two unparallel flaws generated by waterjet cutting with a 0.9 mm nozzle. The cutting speed was adjusted to control the sharpness and length of flaws. Terminology follows the convention introduced by Sagong and Bobet^8^, referring to artificial flaws as “flaws” and resulting fractures as "cracks." The flaw configuration is shown in Fig. 3.

In this study, two artificial flaws were introduced into each specimen: an upper inclined flaw (flaw ①) with endpoints labeled A and B, and a lower flaw (flaw ②) with endpoints labeled C and D. The inclination angle of flaw ① (α₁) was fixed at 45° in all configurations, while the angle of flaw ② (α_2_) varied among five orientations: 0°, 45°, 90°, 135°, and 180°, corresponding to specimen groups S0, S45, S90, S135, and S180, respectively. All flaws were 20 mm in length (2a = 20 mm), and the rock bridge between them was 15 mm long (2b = 15 mm), with an angle β = 45°. In total, more than 32 specimens were prepared and tested. For each flaw configuration, three specimens were tested to ensure result reproducibility. Additionally, two intact specimens without pre-existing flaws (labeled SI-1 and SI-2) were tested under monotonic loading to serve as references for comparison with the flawed specimens. To systematically track testing conditions, each specimen was labeled according to its flaw configuration and loading type. For example, “S0-1-M” denotes the first specimen with α_2_ = 0° tested under monotonic loading, while “S0-1-C” indicates the same flaw geometry tested under cyclic loading. Table 2 summarizes all specimen codes and their corresponding loading parameters.

**Table 2:** Abbreviations for the specimens with cracks under uniform and cyclic loading.

Monotonic loading					Cyclic loading					
Sample	α_2_°	H (mm)	W (mm)	T (mm)	Sample	α_2_°	H (mm)	W (mm)	T (mm)	σ_max_
S0-1-M	0	159.3	80.2	29.3	S0-1-C	0	160.1	80.1	29.9	75%
S0-2-M	0	158.4	79.8	29.8	S0-2-C	0	159.6	80.0	29.5	80%
S0-3-M	0	159.6	79.1	29.2	S0-3-C	0	190.0	79.8	28.7	85%
S45-1-M	45	157.1	77.9	30.1	S45-1-C	45	158.6	79.2	30.1	75%
S45-2-M	45	158.7	78.5	31.1	S45-2-C	45	159.4	78.4	29.4	80%
S45-3-M	45	159.2	80.1	30.2	S45-3-C	45	157.3	79.3	29.7	85%
S90-1-M	90	158.6	80.0	29.7	S90-1-C	90	158.8	80.2	28.9	75%
S90-2-M	90	158.2	79.5	29.9	S90-2-C	90	158.9	80.0	29.6	80%
S90-3-M	90	158	79.4	28.8	S90-3-C	90	159.7	81.1	30.3	85%
S135-1-M	135	157.9	79.9	30.3	S135-1-C	135	159.7	80.4	30.0	75%
S135-2-M	135	158.8	79.3	30	S135-2-C	135	159.1	79.8	30.2	80%
S135-3-M	135	159.1	78.9	29.4	S135-3-C	135	158.7	79.4	29.8	85%
S180-1-M	180	157.5	79.4	29.6	S180-1-C	180	157.8	79.7	29.5	75%
S180-2-M	180	158	79.5	28.7	S180-2-C	180	157.6	79.4	28.7	80%
S180-3-M	180	158.9	79.1	29.5	S180-3-C	180	159.1	78.3	30.4	85%
SI-1	Intact	158.4	80.1	30	–	–	–	–	–	–
SI-2	Intact	159.1	78.8	31.1	–	–	–	–	–	–

**Fig. 4:**
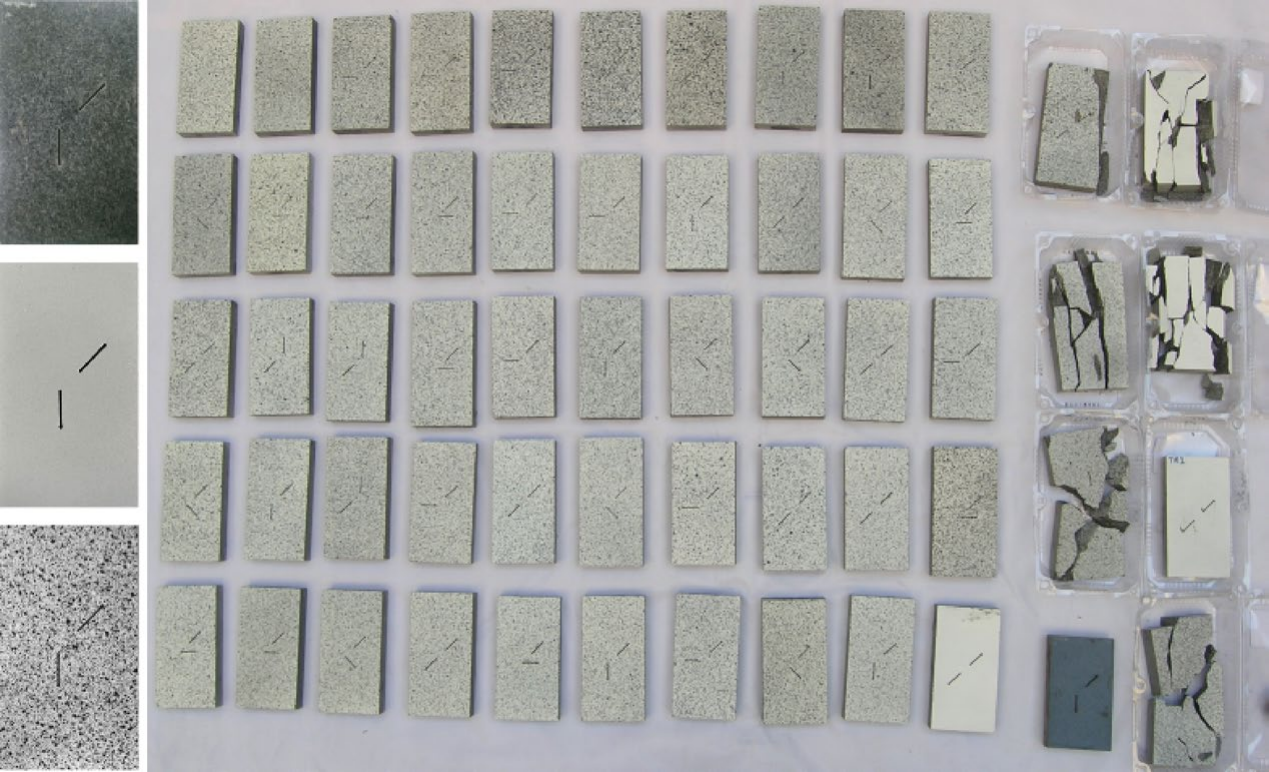
The applied black and white pattern on the specimens and all the specimens in one frame.

Following flaw preparation, all specimen surfaces were polished to ensure uniformity and minimize surface irregularities. A high-contrast black-and-white speckle pattern was applied to facilitate full-field strain measurements using DIC. This pattern was generated by first spraying a uniform layer of matte white paint, followed by the application of a fine distribution of black speckles. Figure 4 illustrates the speckle pattern and all of the prepared specimens.

## Results and discussion

To ensure a systematic and coherent analysis, the results of this study are presented in two main sections based on the loading type: monotonic and cyclic. Within each section, the influence of flaw geometry is assessed with respect to crack types and their propagation paths, crack initiation mechanisms, growth sequences, coalescence patterns, and the overall mechanical properties and strength of the specimens.

### Crack type, propagation path, and deviation

#### Monotonic loading

**Fig. 5:**
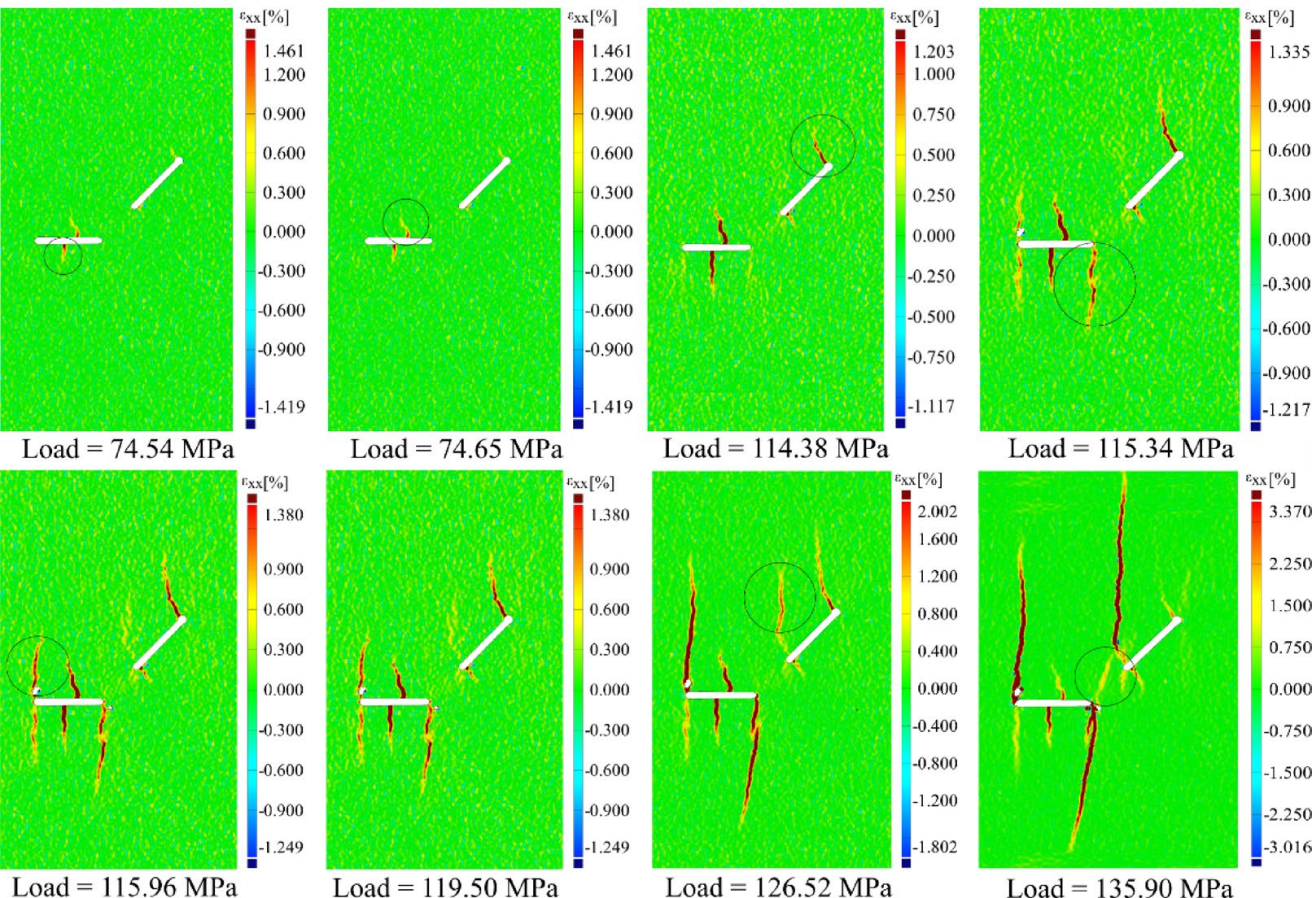
The contour of horizontal local strain in specimen S0-1-M under monotonic loading.

Figure 5 illustrates the horizontal local strain contours of specimen S0-1-M at various stages, from the initiation of loading until final failure. Due to the high number of tests, only the results of this specimen are presented here as representative and are compared with those of other specimens. Initially, as shown in Fig. 5, two primary cracks initiated from the center of flaw ②, propagating both upward and downward. This behavior is likely due to the flaw’s approximate 1 mm thickness, which, under increasing compressive stress, causes slight bending at its center. This bending enhances tensile stress in the middle of the flaw, triggering crack initiation at that location, rather than at the flaw tips, where cracks are typically expected. This central crack initiation aligns with findings from Aliabadian et al.^30^ and Liu et al.^24^, who observed similar behavior in 0° flaws. Their results suggest that maximum tensile stress may shift toward the flaw center. In contrast, other researchers, such as Li et al.^9^ and Park and Bobet^11^, reported tip-initiated cracks. Interestingly, Yang^29^ did not observe such central cracks in sandstone specimens with similar flaw geometries and even thicker flaws (2 mm), possibly due to material differences or limitations in visual detection. However, Liu^24^ confirmed this phenomenon in granite specimens with a single horizontal flaw.

Similar center-initiated cracks were observed in S180 specimens, but differences in geometry altered their propagation paths. In S180, the upper crack deviated from its original path and connected with point B of flaw ①, leading to coalescence. The lower crack also extended more significantly than in the S0 geometry. While both S0 and S180 include a horizontal flaw beneath an inclined one, the degree of overlap between flaws is greater in S180. This increased overlap intensifies the local stress concentration around flaw ②, enhancing crack propagation from the center.

As the applied stress increased, wing cracks initiated at both tips of flaw ① (points A and B). However, only the wing crack at point A exhibited substantial propagation, while the one at point B remained relatively inactive. A similar asymmetric growth pattern was observed in the S45 specimens, where the wing crack originating from point B also failed to propagate significantly. Interestingly, when the second flaw was rotated to 90°, the wing crack at point B displayed limited extension. This behavior can be attributed to the orientation of the 90° flaw, which, being aligned with the loading axis, generated minimal stress concentration and had negligible interaction with the primary flaw. As a result, the stress field surrounding flaw ① intensified, particularly near point B, promoting wing crack propagation. In S135 and S180, the wing crack at point B showed even more pronounced growth. In the S180 configuration, this crack extended sufficiently to coalesce with the adjacent flaw. The enhanced propagation observed in these geometries is likely due to the increased geometric overlap between the flaws, which amplifies the stress concentration both between the flaws and at their tips, thereby facilitating crack coalescence.

Following the initiation of wing cracks, secondary cracks began to propagate in specimen S0-1-M. These originated near the flaw tips, both above and below flaw ②, as well as above flaw ①. In geometries S0 and S45, a secondary crack was observed growing upward from point B of flaw ①, whereas this crack did not appear in the other configurations. Notably, no downward-propagating cracks from point B were observed in either S0 or S45. However, when the angle of flaw ② was adjusted to 90°, an anti-wing crack initiated near point B, propagating downward toward the flaw tip. In the S135 geometry, this anti-wing crack facilitated coalescence between the two flaws, while in the S180 geometry, it was completely absent. Instead, only a wing crack grew from this point in S180, leading to flaw coalescence. These observations clearly demonstrate how variations in flaw geometry can influence not only the presence or absence of crack growth from a specific point but also the type and direction of crack propagation. This behavior is entirely attributed to complex changes in the local stress field induced by different flaw configurations.

Just as changes in flaw geometry affected the path of primary cracks, the same was observed for secondary cracks. In nearly all tested geometries, an anti-wing crack grew from near point A of flaw ①, propagating parallel to the loading direction and almost reaching the bottom surface of the specimen by the final stages of failure. However, in the S180 crack geometry, this crack deviated from its path as it approached point D of flaw ②. In two specimens of S180 geometries, it even connected to point D, which is outside the rock bridge area. This clearly indicates that the stress concentration at point D of flaw ② influenced the stress field at the tip of the crack growing from point A of flaw ①. The change in the path of a crack growing from an inclined flaw upon reaching a horizontal flaw is due to the shielding effect of the horizontal flaw on the stress field of the crack growing from the other flaw. This phenomenon, the shielding effect, was also observed by Wei^17^.

#### Cyclic loading

**Fig. 6:**
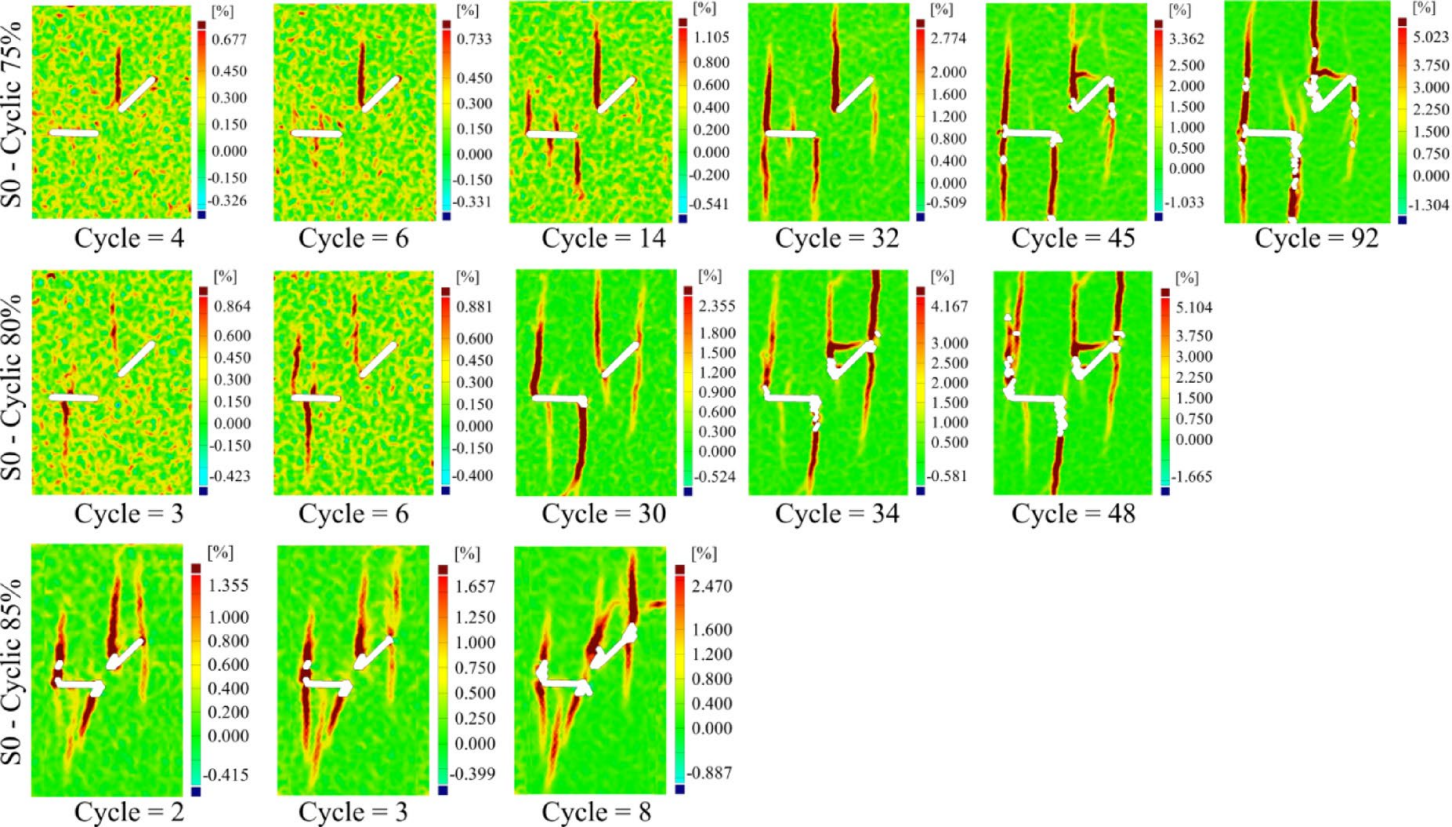
The contour of the major local strain in specimen S0-1-C under cyclic loading in three different amplitudes, 75, 80, and 85%.

Figure 6 illustrates the crack growth in the S0 geometry under cyclic loading, as shown through contour plots of the major local strain (the maximum local strain in both X and Y directions) across different loading cycles. To avoid redundancy and excessive visualization, only the results for the S0 specimens under three different loading amplitudes (75%, 80%, and 85% of the peak load) are presented, which are then compared with the results of other geometries.

Overall, the final crack patterns observed in S0 specimens under cyclic loading relatively resemble those under monotonic loading, although some differences in details are evident. Cracks initiating from the center of the flaw ② typically appear in early cycles due to local bending in the midsection, like monotonic loading, and are considered primary cracks. As the load amplitude increases, the lower crack emerging from this flaw experiences sudden and significant propagation, indicating a strong dependence of the crack growth rate and extent on both the type of loading and the loading amplitude. This can be attributed to a sudden rise in localized stress intensity at the crack tip.

In the case of S180, which features a higher degree of overlap between flaws, a noticeable change in the upper crack’s path is observed. The crack redirects and ultimately connects the two flaws. Simultaneously, the lower crack exhibits further propagation, consistent with monotonic loading results.

Under monotonic loading in the S0 geometry, a wing crack initiated from point A of flaw ①, while another wing crack merely nucleated at point B without propagation. Interestingly, under cyclic loading, no wing crack initiated from flaw ① at any load amplitude. However, similar wing cracks were observed in other geometries under both loading types. This suggests that the absence of wing cracks in S0 under cyclic conditions may result from the complex interplay between loading type and flaws configuration.

As the number of loading cycles increased, cracks similar to those seen in monotonic loading also appeared under cyclic conditions. For instance, anti-wing cracks initiated from both the upper and lower tips of flaw ① and flaw ②. Similar to monotonic loading, no anti-wing crack from point B of flaw ① propagated downward in the S0 and S45 geometries, while such cracks were observed in S90. In this area of the specimen, in S135, an anti-wing facilitated the coalescence of the flaws, and in S180, a single wing crack was initiated from point B.

As in the monotonic case, changes in flaw geometry not only affected the paths of primary cracks but also influenced the propagation behavior of secondary cracks under cyclic loading. For instance, the anti-wing crack initiated near point A of flaw ① in geometries S0, S45, and S90 propagated vertically in line with the loading direction towards the bottom of the specimen. As the angle and overlap increased, this crack deviated toward point D of flaw ②. A particularly notable case was observed in S135. Under monotonic loading, the anti-wing crack grew linearly without connecting to the second flaw. However, under cyclic loading at 75% and 80% of the peak load, the crack path shifted and eventually merged with point D of flaw ②. This behavior is likely driven by the repeated loading and unloading cycles at lower amplitudes, which create transient stress fields with directional bias toward neighboring flaws, facilitating gradual stepwise crack deflection. At higher amplitudes (85%), the crack grew too rapidly for deflection to occur and instead followed a straight trajectory.

Closer inspection of Fig. 6 reveals that, at 75% and 80% loading amplitudes, horizontal cracks formed in cycles 45 and 34, respectively. These cracks extended from the initiation point of the anti-wing crack at point B toward point A of flaw ①. Notably, these horizontal cracks were observed exclusively under cyclic loading and will henceforth be referred to as “cyclic cracks.” These cracks in S0 consistently initiated from the same region and appeared at lower loading amplitudes. Their formation appears to be a result of repetitive loading and unloading on the specimen surface, causing relative motion between opposing crack faces of the anti-wing crack grown from point B at flaw ① upwards of the specimen. Occasional interlocking of these surfaces may lead to localized tensile stresses, which eventually promote horizontal crack propagation. Observations indicate that these cyclic cracks are highly dependent on the number of load cycles applied. As the loading amplitude increases, reducing the number of cycles sustained by the specimen, such cracks no longer appear. A similar behavior was noted in S135, where cyclic cracks systematically formed at specific locations at 75% and 80% loading amplitudes but disappeared at 85% amplitude. In other geometries, cyclic cracks were generally absent, indicating that their formation appears closely tied to both the number of applied cycles and the specific geometrical arrangement of the flaws. Overall, these trends suggest that the formation or absence of cracks at a given location, their type, and the propagation path depend on both flaw geometry and loading type. This leads to differences in the overall crack pattern in certain geometries, such as S0 and S135.

### Sequence of crack development

#### Monotonic loading

**Fig. 7:**
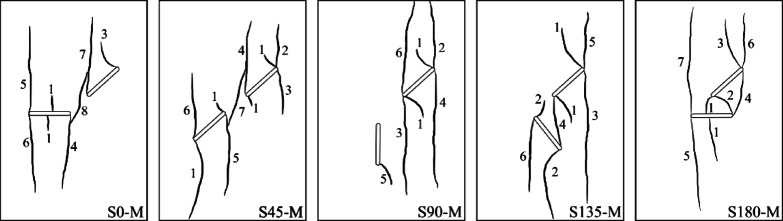
Schematic crack growth sequence of different geometries.

As illustrated in the S0-1-M specimen (Fig. 5), wing cracks formed as primary cracks and after them anti-wing cracks as secondary cracks. This pattern was generally consistent across all geometries. However, the origin of the primary cracks (whether from flaw ① or flaw 2) varies depending on the specific geometry. Figure 7 presents a schematic representation of the observed crack patterns obtained using the DIC technique, aimed at clarifying the crack growth sequence. In this picture, cracks were numbered based on their formation sequence through loading. It is important to note that each geometry was tested three times to ensure reproducibility, and Fig. 7 shows only one representative image of the crack patterns from different specimens.

This figure demonstrates that in the S0 and S180 geometries, the primary cracks initially initiate from flaw ②, while in S90 and S135, they first originate from flaw ①. In the S45 geometry, primary cracks appear simultaneously from both flaws. It appears that when the angle of flaw ② is zero (as in S0 and S180), the primary cracks tend to propagate due to bending at the midpoint of flaw ②, requiring less stress to activate compared to the wing cracks initiated from flaw ①. In the S45 specimens, the simultaneous initiation of wing cracks from both flaws is likely due to the parallel orientation of the flaws, which leads to nearly equal stress concentrations around them.

Regarding the sequence of secondary crack propagation from flaws ① and ②, which was related to the anti-wing cracks, Fig. 7 shows that in all geometries, secondary cracks initially initiate from flaw ①, except in the S0 geometry, where they start from flaw ②. When comparing this behavior in the S0 specimen with the most similar geometry, S180 (which only differs in the degree of overlap between the flaws), it appears that in S180, the overlap created by flaw ① has a shielding effect on flaw ②. As a result, the stress required to initiate secondary cracks from flaw 2 increases, causing them to form at later stages.

#### Cyclic loading

**Fig. 8:**
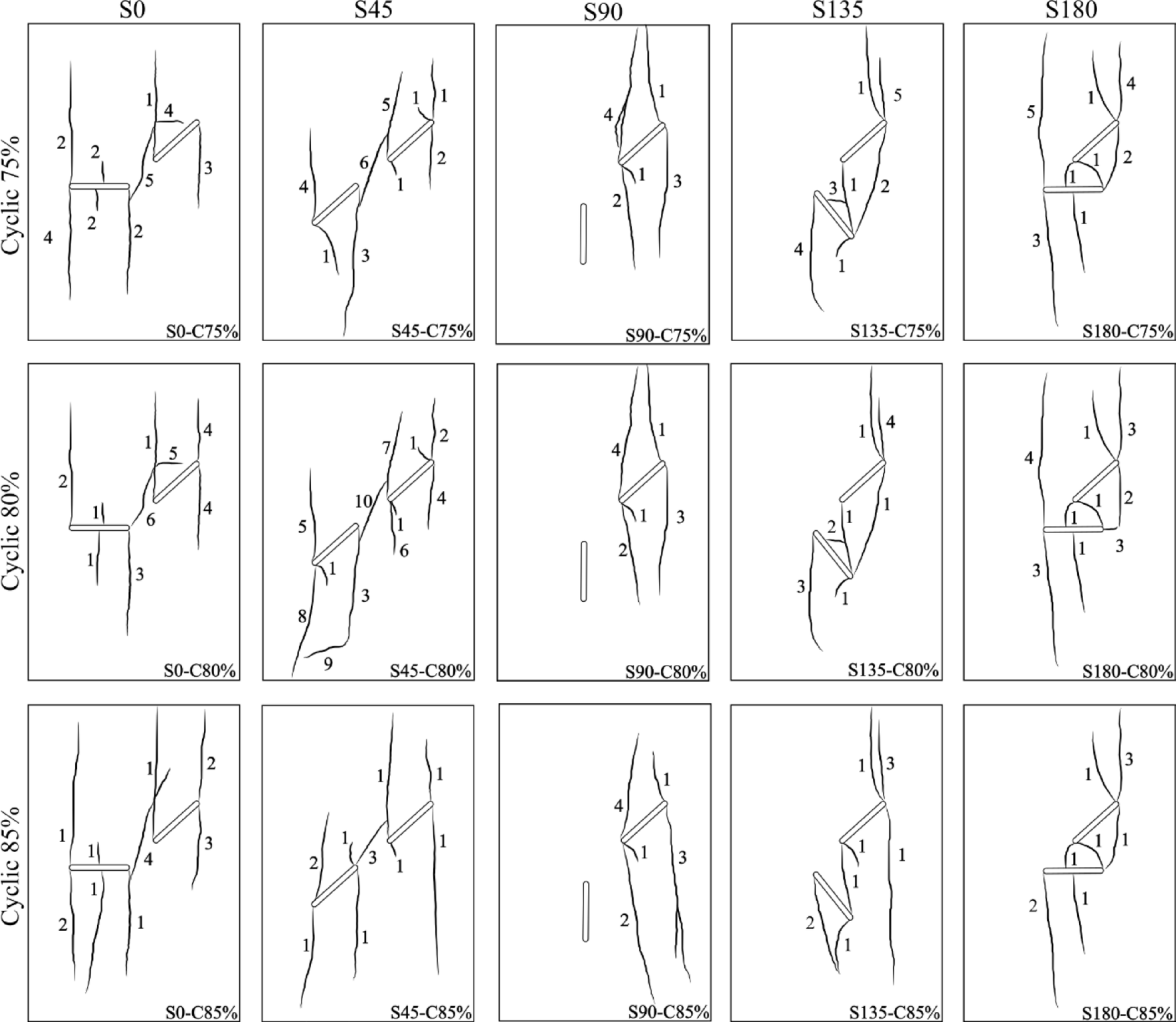
Sequential timeline of crack propagation across various flaw geometries and different cyclic loading amplitudes.

Figure 8 illustrates the chronological sequence of crack growth for different geometries under three cyclic loading amplitudes, 75%, 80%, and 85% of each specimen’s ultimate strength. A comparison with the crack evolution observed under monotonic loading reveals significant differences in the temporal order of crack development between the two loading conditions. In the S0 specimen at a 75% loading amplitude, the first crack to appear was an anti-wing crack originating near point B of flaw ①. This crack formed during the fourth loading cycle. Subsequently, two additional cracks initiated from the center of flaw ② during the next two cycles. Interestingly, this sequence is reversed under monotonic loading, where the anti-wing crack typically emerges during the later stages of loading. This means that the anti-wing crack, which acted as a secondary crack under monotonic loading, functioned as a primary crack under cyclic loading.

As the load amplitude increases to 80%, both the anti-wing and the central cracks grow almost simultaneously and at a faster rate, appearing in the third cycle. A noteworthy trend emerges: as the load amplitude increases, the number of cracks initiating concurrently also rises. At 85% loading amplitude, up to five primary and secondary cracks emerge simultaneously during the second cycle, exhibiting substantial growth.

These findings suggest that sudden increases in stress amplitude can simultaneously surpass the initiation thresholds for multiple cracks, leading to synchronous crack growth across the specimen. Changes in crack geometry also affect the crack growth sequence.

In S90 geometry, the sequence of crack formation follows the same pattern as under monotonic loading—primary cracks develop first, followed by secondary cracks. While S45 geometry showed this same behavior at 75% and 80% loading amplitudes, at 85% amplitude, all seven cracks developed simultaneously within the same loading cycle. However, in geometries with higher overlap, particularly S180, the impact of increased loading amplitude on synchronous crack propagation becomes more pronounced. In this configuration, four and five cracks were observed to grow simultaneously at 80% and 85% loading amplitudes, respectively. These results highlight that in highly overlapping geometries, increasing the loading amplitude significantly enhances the simultaneous initiation and propagation of both primary and secondary cracks.

### Initiation mechanism and coalescence pattern

#### Monotonic loading

By analyzing horizontal displacement and strain plots, we can accurately examine and compare the created cracks, their propagation paths, and their chronological sequence. However, the exact mechanism behind the initiation and growth of these cracks remains a question: are they purely tensile (Mode I), purely shear (Mode II), or a combination of these two modes?

**Fig. 9:**
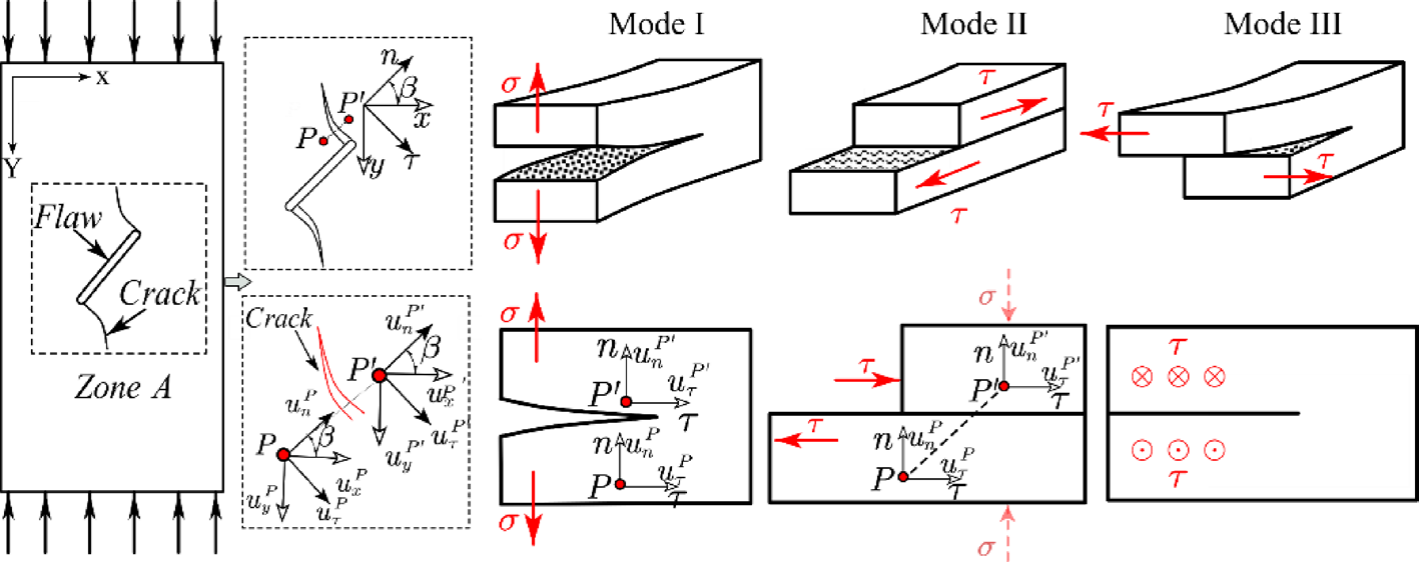
Liu’s method for investigating the nature of crack initiation and propagation^24^.

Historically, visual observation, often using tools like high-speed cameras, was the common method for investigating this phenomenon. In this approach, cracks were typically categorized based on their fracture surface appearance^11,12,19^. Yet, this method suffered from limited accuracy and reproducibility due to its subjective nature. In contrast, theoretical models based on Linear Elastic Fracture Mechanics (LEFM) offer an analytical framework for predicting crack initiation conditions, but their simplified assumptions mean they don’t always align with real-world scenarios^18^. As mentioned earlier, the DIC technique has emerged as a non-contact and precise tool for quantitative analysis of the crack initiation process^24,26^. Notably, Liu et al.^24^ presented a novel method for determining Mode I, Mode II, or mixed-mode fracture types by analyzing displacement components parallel and normal to the crack direction. This approach offers significantly higher accuracy and objectivity compared to visual methods. According to this method, which is described in Fig. 9, after the cracks have grown and their growth path is determined, two points are taken on either side of the crack path such that the line joining these points is normal to the crack growth path.

After defining these two points in the reference image (picture of the specimens before applying load), we determined their displacements in both X and Y directions and calculated their relative displacement. Notably, when a grown crack formed at an angle to the loading direction, the relative displacements normal and tangent to the crack surface could be obtained by calculating angle β and applying the rotation matrix.

Following the determination of relative displacements around the crack, we identified the initiation mode of grown cracks using Liu’s criteria (1) and (2).

1 $${\text{Mode I}}:\left\{ {u_{n}^{{p'}} - u_{n}^{p} > 0~ \cup \left| {u_{\tau }^{{p'}} - u_{\tau }^{p} } \right| = 0} \right\}$$

2 $${\text{Mode II}}:\left\{ {\left| {u_{\tau }^{{p^{\prime}}} - u_{\tau }^{p} } \right| > 0 \cup u_{n}^{{p^{\prime}}} - u_{n}^{p} \le 0} \right\}$$

**Fig. 10:**
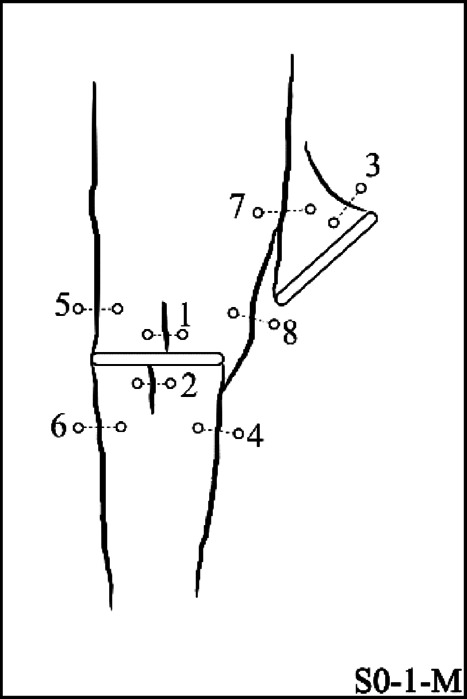
Defined points positioned normal to the crack growth paths observed in the S0-1-M specimen.

In these relations, ${u}_{n}^{p{\prime}}$, ${u}_{n}^{p}$, ${u}_{\tau }^{p{\prime}}$ and ${u}_{\tau }^{p}$ the displacement components are in the normal and shear directions of the propagated crack, and Mode I and Mode II are the failure modes presented by LEFM. It should be noted that Mode III, i.e., the rupture mode, cannot be calculated using this method; therefore, in this paper, Modes I and II in the propagated cracks were investigated. In order to demonstrate the analysis process on different crack geometries in a monotonic loading condition, the following analyses performed on the S0-1-M specimen are presented in detail. To avoid repetition and prolongation of the material, only the results obtained from examining crack growth in other specimens, using the same analytical process, are reported. In Fig. 10, the propagated cracks in specimen S0-1-M are shown, together with two reference points located along the different crack paths.

**Fig. 11:**
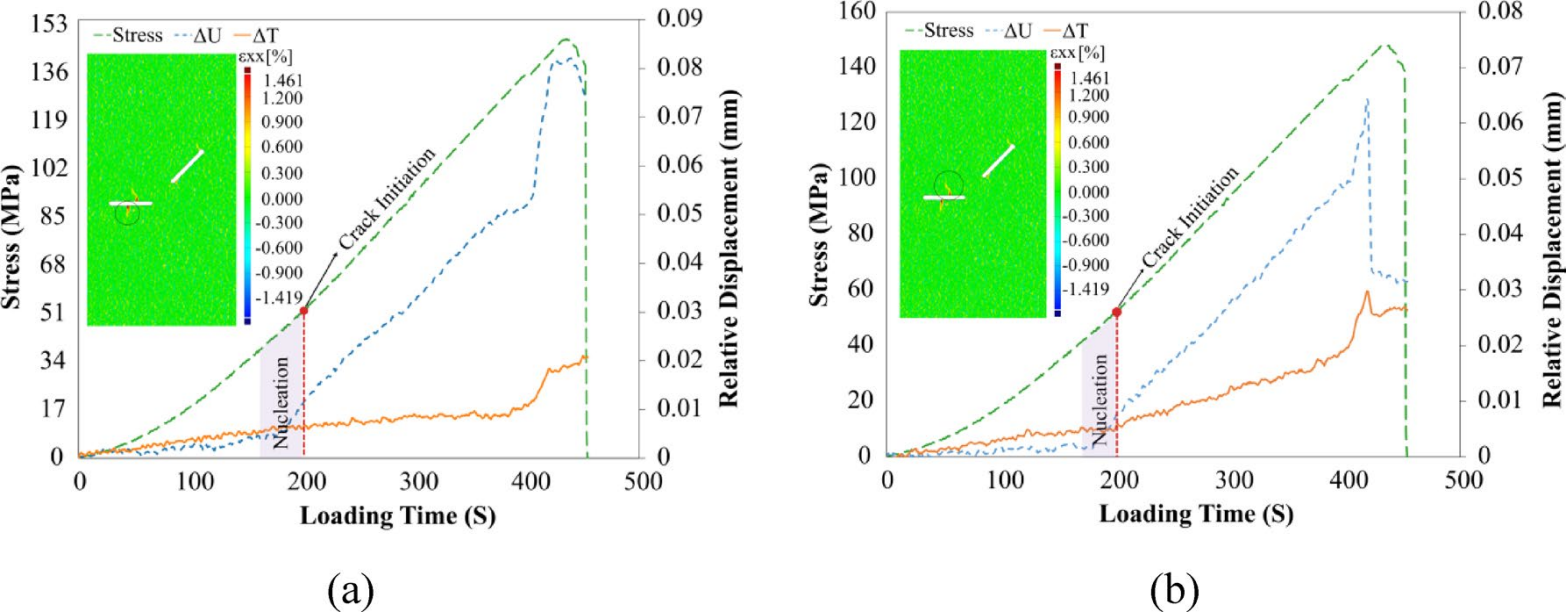
Relative displacement around the primary cracks propagated from the center of the flaw ② along with horizontal local strain contours, (**a**) Crack No. 1, (**b**) Crack No. 2.

These two points were created based on the crack initiation places, which were a total of 8 cracks numbered based on the sequential order from 1 to 8. In the following, the propagated cracks in the specimen were analyzed clearly and separately in the order they appeared from the early stages of crack growth. Figure 11 presents the stress vs. time and relative displacements vs. time diagrams for two points surrounding the initial propagated cracks (cracks No. 1 and 2) from the flaw ② center, along with horizontal strain contours. As observed, these cracks simultaneously appeared at the 216th second of loading on the specimen surface, as indicated by the strain contours in the X-direction on the specimen surface.

In Fig. 11a, the results clearly indicate that the displacement normal to the Crack No. 1, which is circled, exhibits a greater magnitude and a more pronounced increasing trend compared to the shear displacement to it throughout the loading period. This crack was first detected on the specimen surface at frame 216, where the normal and shear relative displacements measured approximately 0.01 mm and 0.003 mm, respectively. A review of the preceding frames (186–216) revealed that the normal displacement rose steadily from around 0.003–0.01 mm, whereas the shear displacement remained essentially unchanged at about 0.003 mm. These findings suggest that, although both displacement components were present before crack initiation, only the normal component exhibited a significant increase. Consequently, the mechanism of the crack initiation mode of Crack No. 1 may be attributed primarily to mode I (tensile) opening, with the shear displacement remaining negligible throughout. Regarding the crack growth mode during loading, the relatively constant trend in shear displacement versus the increasing trend in normal displacement suggests Mode I-dominated growth. These findings were supported by the crack classification studies of Liu^24^, who observed Mode I for this crack. It appears that the sudden increase in the relative displacement normal to the specimen surface is due to its rapid growth at loading step 400. This increase continued until the propagation of secondary cracks. After the secondary cracks began to propagate, the stress concentration on the primary cracks was relieved, which led to a reduction in the crack opening. Crack closure is consistent with DIC studies^20,31^ and observations by Cao et al.^32^ on post-failure closure. However, it does not return to its initial value from loading step 295, which is why it does not fully recover.

The Crack No. 2 emerged on the specimen surface at loading frame 216, with a normal relative displacement of 0.011 mm, roughly twice the shear displacement of 0.005 mm. Unlike the lower crack, which propagated almost from the midpoint of flaw ② (Fig. 11b), this crack began slightly off-center and exhibited mixed Mode I/II characteristics. Although its normal displacement curve rose as steeply as that of the Crack No. 1, the shear displacement curve showed an even sharper incline just before crack formation. This behavior indicates that tensile opening (Mode I) dominated, but in‐plane shear (Mode II) also contributed to crack initiation and growth. Theoretically, treating flaw ② as a beam under bending loads predicts maximum tensile stresses at its midpoint, explaining why the centrally located Crack No. 1 was purely Mode I, whereas the off‐center initiation of the Crack No. 2 naturally produced a combined Mode I/II behavior. Both normal and shear relative displacements of Crack No. 2 showed an initial increase followed by a sharp rise as the secondary cracks developed. Following the expansion of the secondary cracks, these displacements experienced a greater reduction, indicating a more pronounced closure of the crack surface in comparison to the Crack No. 1. For clarity and simplicity, the following abbreviations will be used in the next analysis: Mode I (T), Mode II (S), mixed-mode I/II with tensile dominance (T_S_), mixed-mode I/II with shear dominance (S_T_), and mixed-mode I/II with approximately equal influence of both modes (TS).

**Fig. 12:**
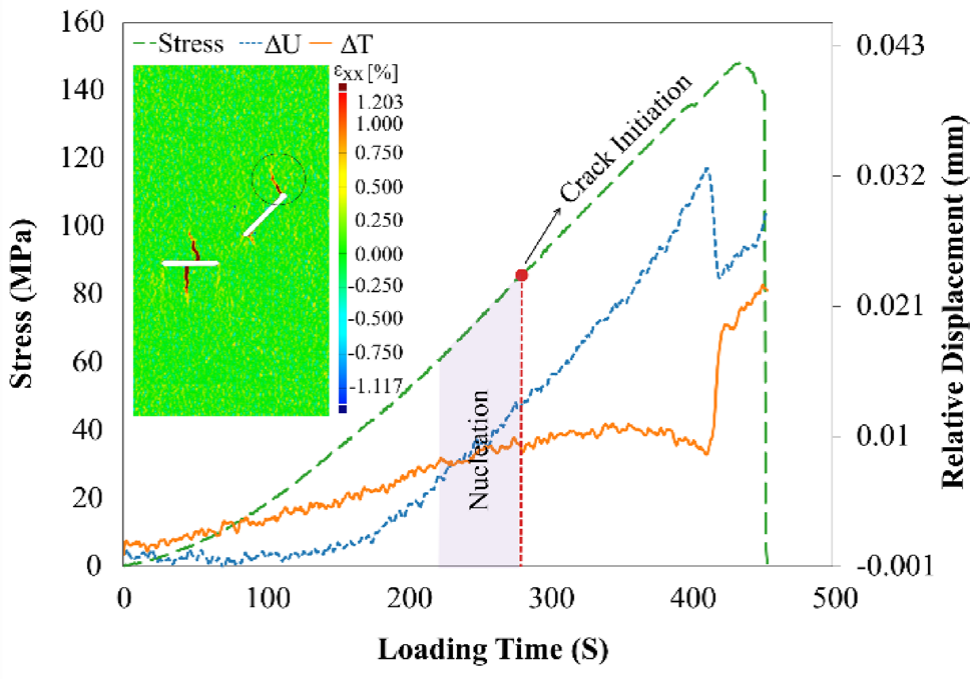
Relative displacement around the Wing crack propagated from the tip of the flaw ① along with the horizontal local strain contour.

The third observed crack (Crack No. 3) in the specimen, referred to as a wing crack, initiated from the tip of flaw ① with an angle of 31° with respect to the normal loading and propagated along a curved-linear path. Figure 12 presents the relative displacement plots and horizontal strain contours associated with the propagation of this crack. As shown in Fig. 12, the observed behavior of this crack is similar to that of the two primary cracks described in the previous stage. Crack No. 3 nucleated from point A in Flaw ① at a load frame of 260. The shear and normal relative displacements were 0.01 mm and 0.009 mm, respectively. These values indicated that this crack belonged to the Ts-mode category, as it exhibited both separation and slippage simultaneously. However, with regard to its propagation mode, it was approximately in a T mode since the shear relative displacement was constant until the loading frame of 410, and then changed its propagation mode to Ts mode until the end of loading.

**Fig. 13:**
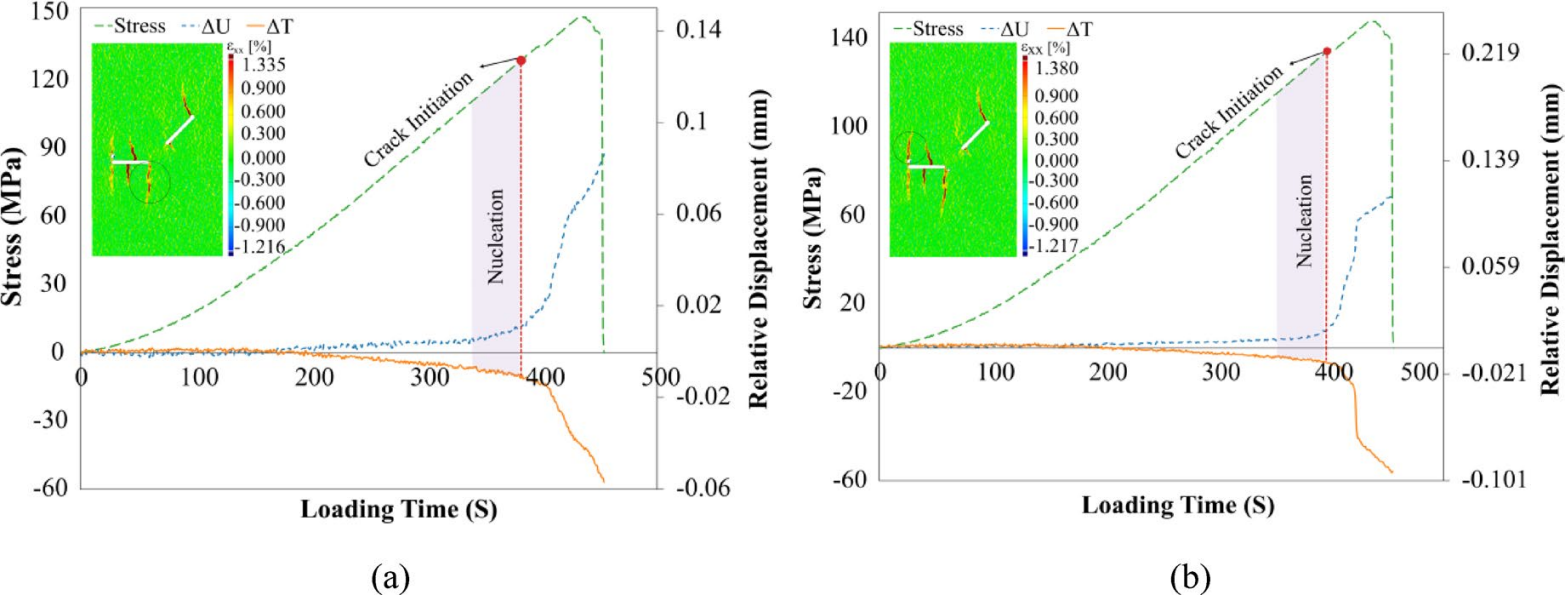
Relative displacement around (**a**): Crack No. 4 and (**b**): Crack No. 5 propagated from flaw ②, along with horizontal local strain contours.

Cracks No. 4, 5, 6, and 7, also referred to as secondary cracks or anti-wing cracks, initiated close to each other, approximately from the vicinity of the tip of flaws ① and ②, and began to propagate with different characteristics. The propagation of these cracks led to a noticeable drop in the stress curve. As shown in Fig. 13 and based on the relative displacement plots of the propagating cracks, it can be observed that Cracks No. 4 and 5, which initiated near flaw ②, one propagating downward and the other upward, exhibited similar trends in relative displacement.

Both cracks exhibited relative displacements of approximately 0.01 mm in the normal and shear directions, but at different load frames (Crack No. 4 at 381 and Crack No. 5 at 390). The displacement behavior at the onset of crack growth suggests that both cracks were governed by the TS mechanism, involving Modes I and II simultaneously. Due to the symmetry in the displacement plots, the contributions of each mode to crack initiation appear equal. In terms of propagation mode, both cracks maintained the TS mode until nearly the end of loading. Liu’s 2020 studies on cracks emanating from a single flaw predominantly classified anti-wing cracks as Mode II fractures. However, in our experimental configuration with dual flaws, these anti-wing cracks demonstrated distinct mixed-mode behavior, with nearly comparable contributions from both Mode I and Mode II components.

**Fig. 14:**
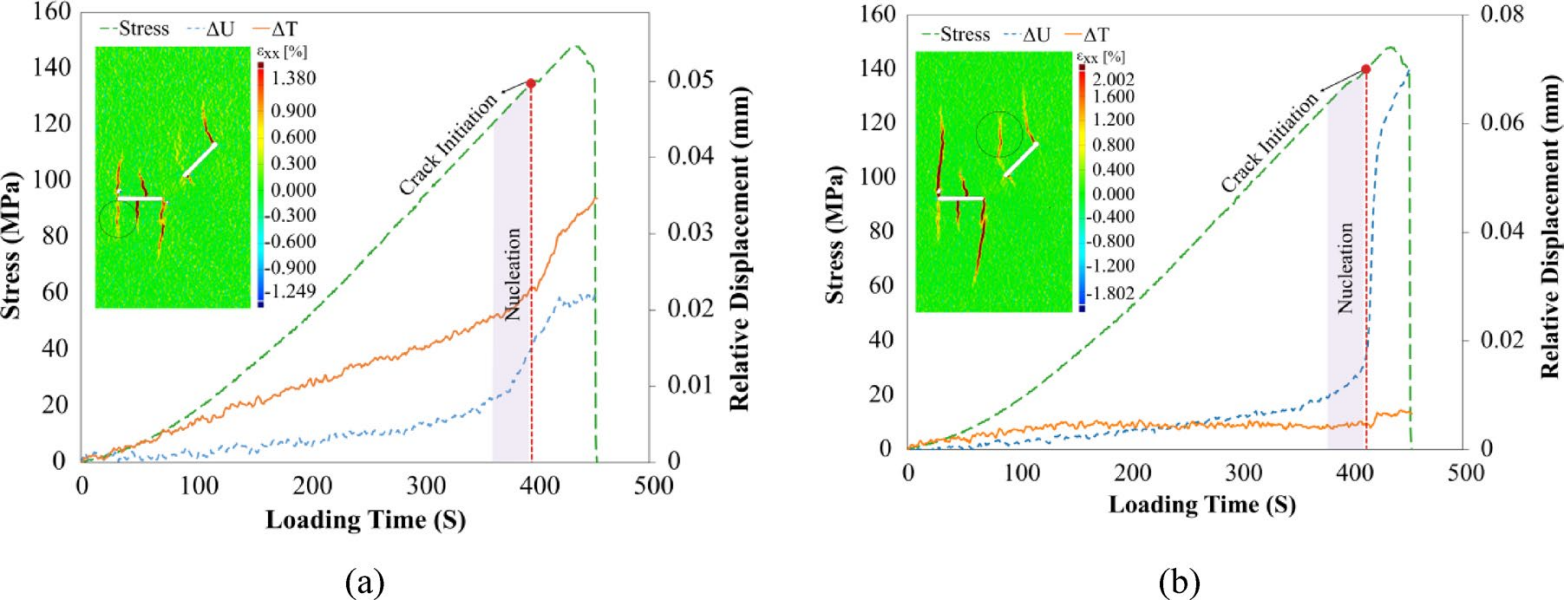
Relative displacement around (**a**): Cracks No. 6, and (**b**): 7 propagated from flaw ② and flaw ①, respectively, along with horizontal local strain contours.

The relative displacement plots of Cracks No. 6 and 7, presented in Fig. 14, indicate that Crack No. 6 (Fig. 14a) is the only crack for which the shear relative displacement across the specimen surface exceeds the normal relative displacement throughout the entire loading process. It nucleated on the specimen’s surface at load frame 391, with normal and shear relative displacements of 0.01 mm and 0.02 mm, respectively. The shear displacement was twice as large as the normal displacement. Based on the displacement behavior at the moment of crack initiation, this crack can be classified as a mixed-mode S_T_ crack, with Mode II being dominant. Although Crack No. 7 (Fig. 14b) is a secondary crack that forms during the final stages of loading (load frame 400), its relative displacement trend resembles that of the primary cracks. Specifically, similar to the primary cracks, a significant increase in normal relative displacement is observed before crack initiation, while the shear displacement shows relatively minor changes. The crack nucleated at the surface, exhibiting normal and shear relative displacements of 0.012 mm and 0.004 mm, respectively. This displacement ratio (3:1) demonstrates Mode I dominance over Mode II. Therefore, this crack is classified as a mixed-mode T_S_ crack, with Mode I being dominant.

**Fig. 15:**
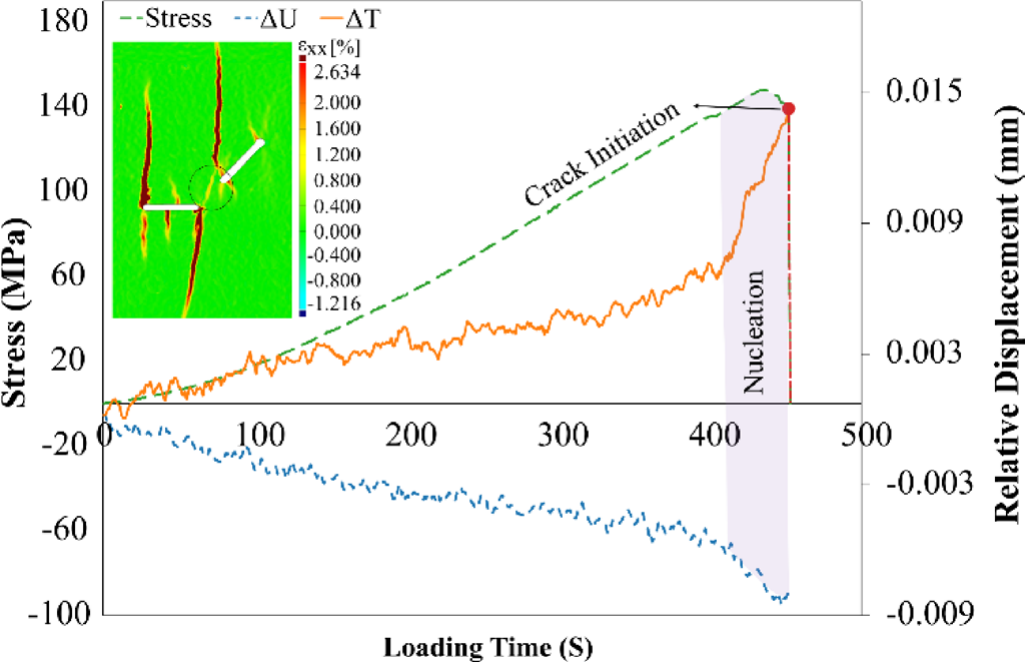
Relative normal and shear displacement profiles across crack No. 8, responsible for the coalescence between flaws, along with a horizontal local strain contour.

Coalescence cracks (Crack No. 8) in this specimen occurred at the final moments and at a very high rate. Due to the image acquisition rate of 1 frame per second, it was not possible to fully capture the propagation of crack No. 8. However, the initiation site of this crack was identifiable through the noticeable strain developed on the surface. After the final failure of the specimen, it was observed that a crack had propagated near the initiation point of crack No. 7 toward a region near point C from flaw ②. Physical inspections and cross-sectional observations indicated that this crack exhibited a shear (S) mode. Based on the identified crack path on the surface, two points on either side of the crack were selected for digital image analysis, and the relative displacements parallel and normal to the crack were calculated before final failure. The results are illustrated in Fig. 15. This figure shows that both displacement components increased significantly and simultaneously before the final failure. The negative trend of normal relative displacements suggests that the two faces of the crack surface are moving closer together while slippage occurs on the surface. This indicates that the crack is propagating in an S-mode.

**Fig. 16:**
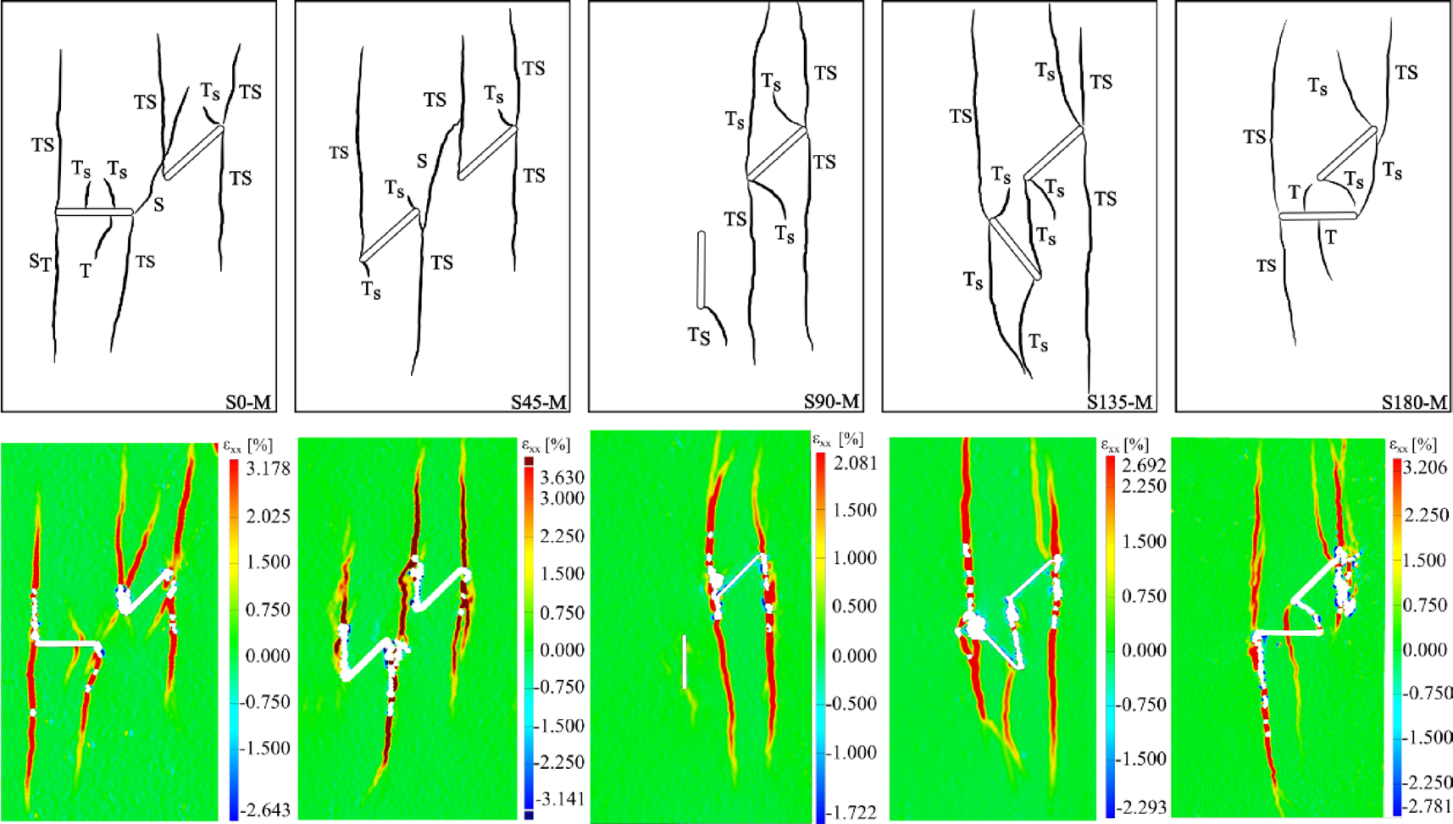
Final fracture patterns showing crack initiation nature and horizontal local strain distribution under monotonic loading.

Based on the conducted analyses, the mechanisms of crack initiation and propagation for all cracks developed under different flaw geometries were identified. Figure 16 presents the final fracture patterns for each configuration, along with the corresponding crack initiation mechanisms adjacent to each propagated crack.

This figure presents plots of the horizontal strain of the specimens at the final fracture moment, along with a schematic representation of the cracks grown from the flaws and their initiation modes in various geometries. To avoid an excessive number of figures, only one out of the three tested specimens, which is representative of all grown cracks, is displayed. As observed in Fig. 16, a change in flaw geometry directly influences the crack initiation mechanism. This alteration is particularly evident in the ligament region between the two flaws. For instance, in the S90 geometry, the anti-wing crack growing from flaw ① point B exhibits a TS nature (an equal combination of tension and shear). However, in the S135 specimen, this same anti-wing crack takes on a Ts nature, meaning tension plays a more significant role in crack initiation compared to shear. Furthermore, the primary crack grew from the center of flaw ①, which showed Ts mode in the S0 specimen, and presented T mode in the S180 specimens. This difference indicates that the rotation of flaw ② alters the stress field around the flaws, highlighting the complexity of stress in the ligament region. Furthermore, it was determined that in areas where the stress field is nearly uniform across all specimens (regions outside the ligament), the nature of the crack initiation mechanism remains unchanged. A closer examination of the specimens revealed that in the S0 and S180 specimens, which are angularly similar but differ in overlap, the crack propagating downwards from point D exhibits two different modes: TS and S_T_. This distinction is due to the influence of flaw overlap on the initiation mechanism of this crack, rather than a direct effect of the flaw geometry.

In studying how changing flaw geometry affects coalescence between two flaws, results showed that among the five geometries examined, specimens S0 and S45 experienced indirect coalescence, while S135 and S180 exhibited direct coalescence. Notably, no coalescence was observed between the two flaws in the S90 geometry. In the S0 and S45 geometries, coalescence occurred via a crack with an S mode (pure shear). Conversely, in the S135 and S180 geometries, the crack responsible for coalescence had a Ts composite mode, meaning the tensile component was more prominent. It is worth noting that in S180 geometries, coalescence occurred directly through three different cracks, which exhibited T and Ts modes, whereas in the S135 specimen, only one crack was responsible for coalescence. These findings indicate that as the flaw geometry shifts from a non-overlapping to a more overlapping configuration, the role of tension in the coalescence process becomes more pronounced, and coalescence occurs directly. The absence of coalescence in the S90 specimen is noteworthy. Due to the angle of flaw ② in this geometry, stress concentration was primarily on flaw ①. This resulted in flaw ② having minimal participation in the coalescence process, causing the specimen to behave as if only a single flaw were present.

#### Cyclic loading

Similar to the approach used for identifying crack initiation modes under monotonic loading, the same analysis was applied to the cyclic loading conditions. In what follows, crack mode evaluations are presented for the S0 geometry under three different loading amplitudes (75, 80, and 85% maximum monotonic strength in each geometry), and the results are compared with those from other geometries. Overall, it was observed that the cracks formed under cyclic loading exhibited strong similarities in terms of crack initiation mechanism to those developed under monotonic loading.

**Fig. 17:**
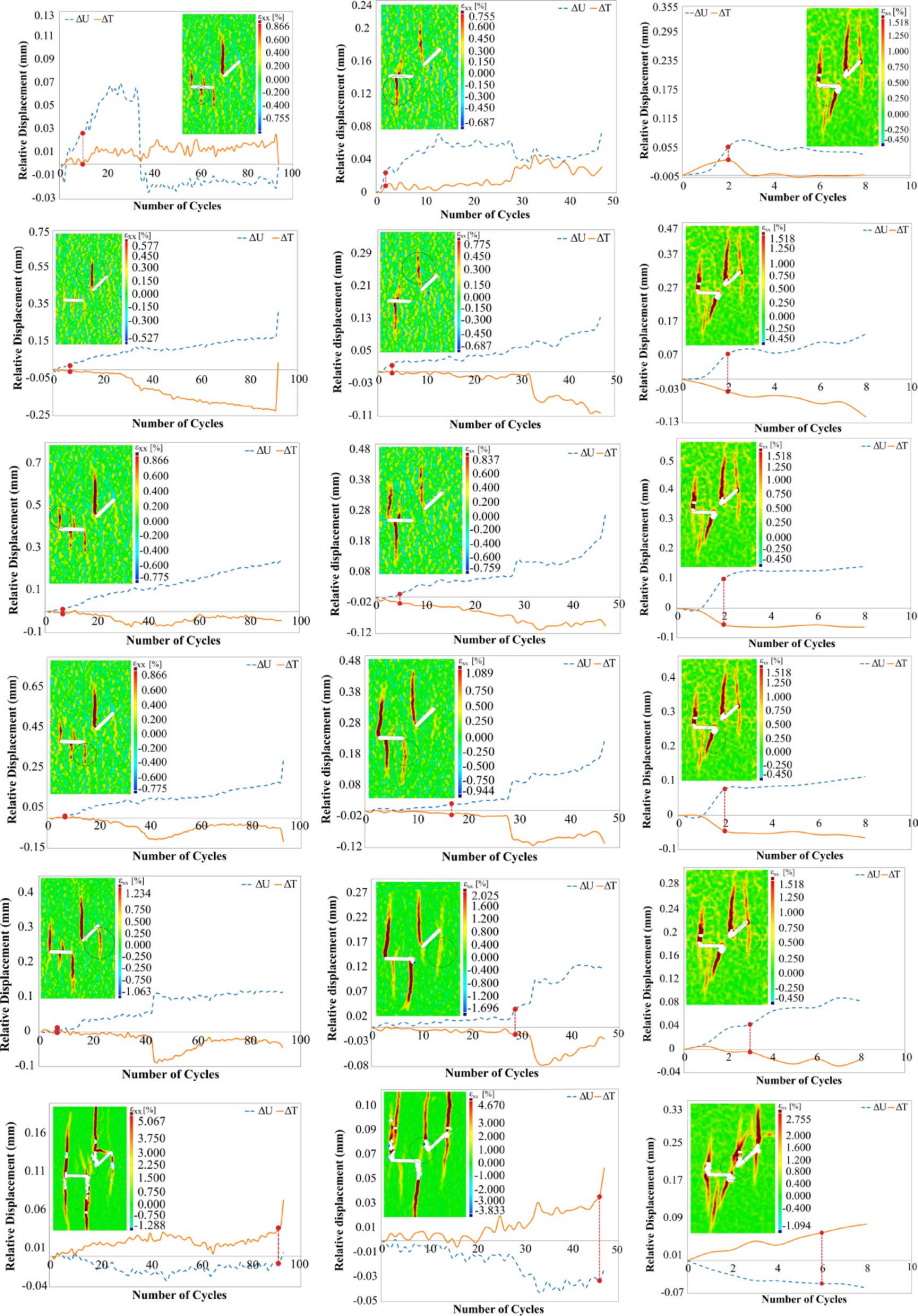
Relative displacements and horizontal local strain for S0 under three cyclic loading amplitudes.

After defining two reference points on either side of each grown crack, their relative displacements during the loading and unloading phases were calculated. Since the objective was to compare crack behavior under pressure, only the data from the maximum loading stage were analyzed, and displacements during unloading were excluded. Moreover, only the cracks that were commonly observed under all three loading amplitudes were considered for analysis. Figure 17 presents the shear and normal relative displacements around the cracks, along with horizontal local strain contours for visualizing crack positions and paths.

The relative displacement analysis at the onset of cracking indicated that all cracks initiated from a given location exhibited the same initiation mode in three different amplitudes, similar to that observed under monotonic loading. For example, both upward and downward cracks from flaw ② initiated in mode T_S_, but their propagation modes differed under monotonic loading. In the S180 geometry, the crack connecting the center of flaw ② to point B of flaw ① exhibited pure tensile (T) fracture behavior. This suggests that the geometry of flaws significantly influences fracture mode, likely due to changes in the local stress field induced by geometric variations.

**Fig.18:**
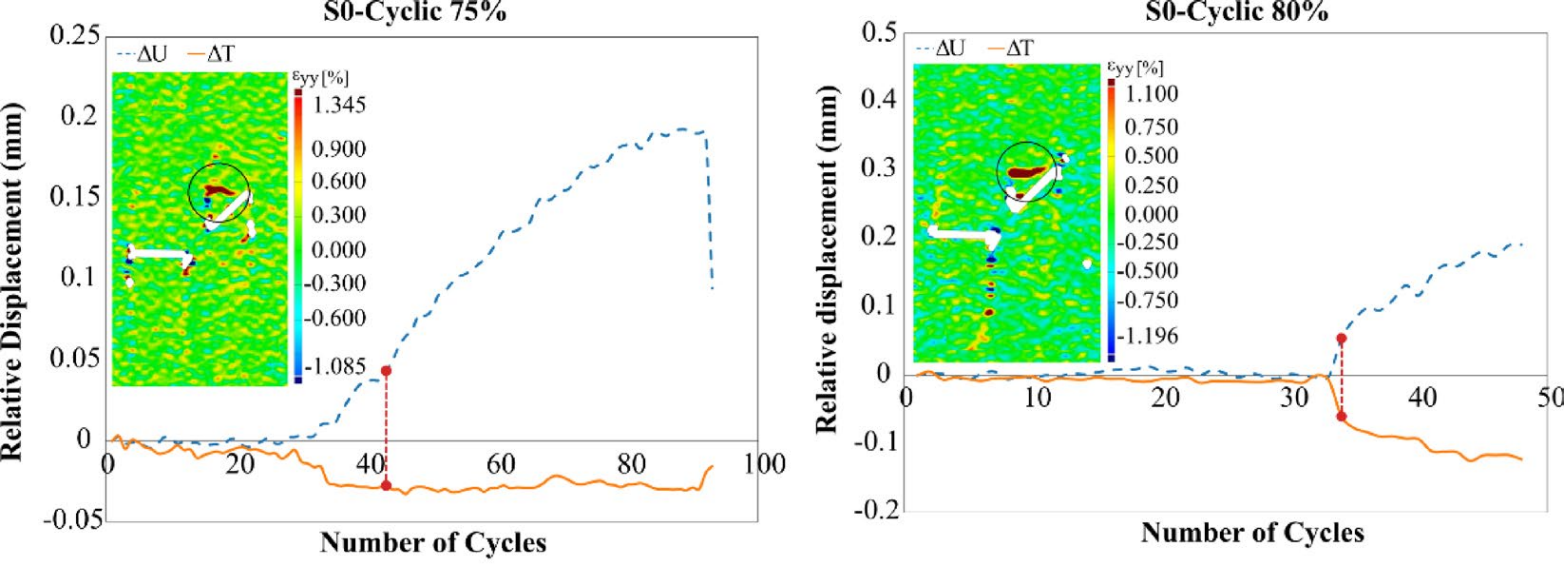
Relative displacement profiles, along with normal local strain contours, for cyclic cracks in S0 specimens subjected to cyclic loading.

The so-called “cyclic cracks” that appeared in the S0 geometry at 75% and 80% loading amplitudes were not clearly visible in the horizontal local strain contours. Therefore, these cracks were examined using normal local strain contours, as shown in Fig. 18, alongside plots of relative shear and normal displacement. The analysis revealed that the crack at 75% amplitude exhibited a nearly pure tensile (T) mode, while the one at 80% showed a mixed-mode T_S_ behavior. The cyclic cracks observed in the S135 geometry also followed a T mode, and in this case, the flaw geometry did not affect the fracture mode.

**Fig. 19:**
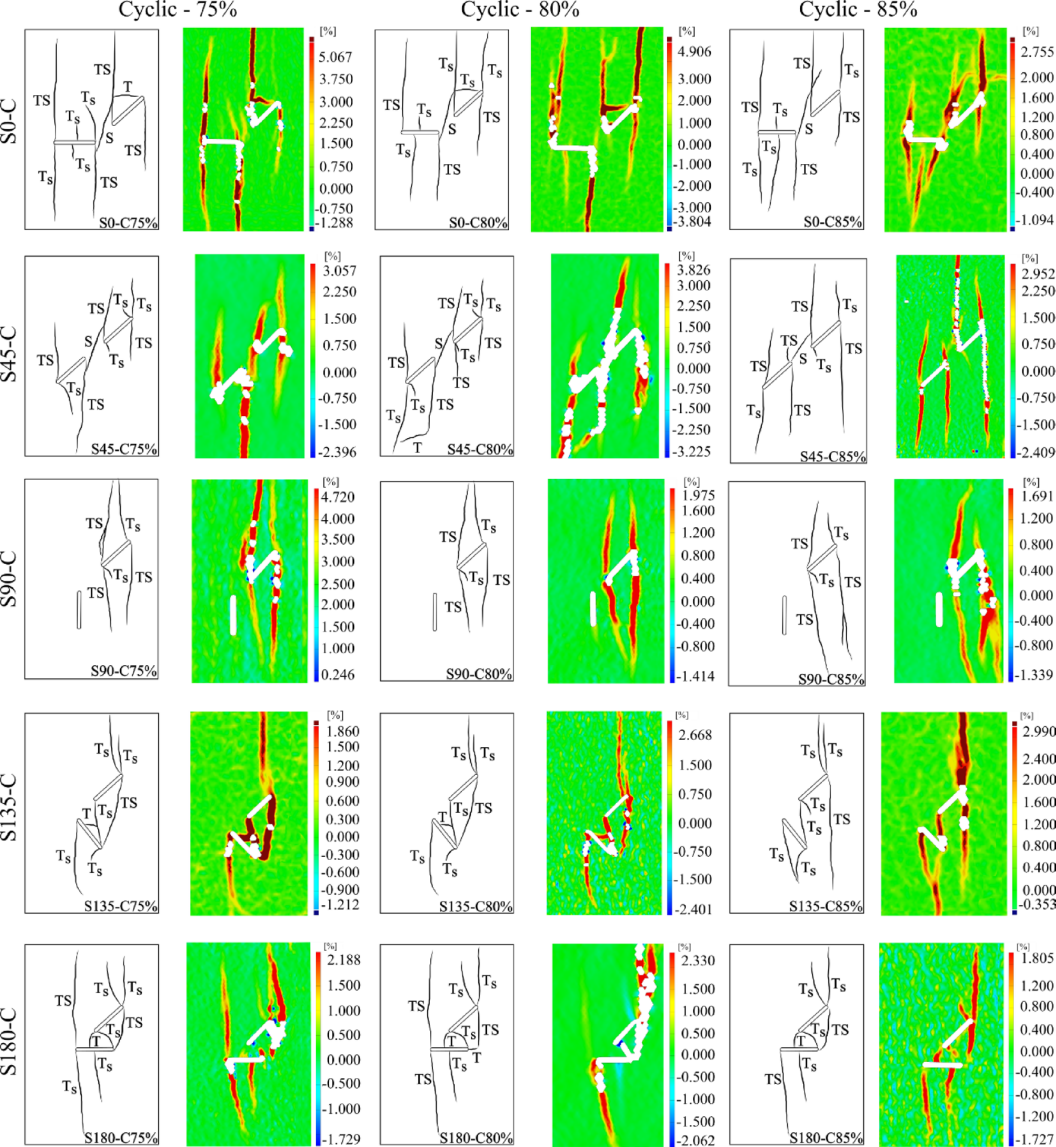
Final fracture patterns showing crack initiation nature and major local strain distribution under Cyclic loading.

Overall, the analysis revealed that, similar to monotonic loading, in geometries S0 and S45, crack coalescence across all cyclic loading amplitudes occurred indirectly via the growth of an S-mode crack. In contrast, S90 did not show any coalescence, consistent with the monotonic case. For overlapping geometries like S135 and S180, direct coalescence between cracks was observed. Hence, it can be concluded that the loading type (monotonic vs. cyclic) had no significant effect on the crack initiation mechanism or the nature of crack coalescence. Figure 19 displays schematic diagrams of the final crack pattern, along with their associated initiation mechanisms, and the major local strain contours for each of the three loading amplitudes. Note that these contours correspond to the last loading stage; thus, some cracks that grew during earlier stages may not be visible, as they subsequently closed and left no trace in the final contour image.

### Mechanical properties and strength characteristics

#### Monotonic loading

**Fig. 20:**
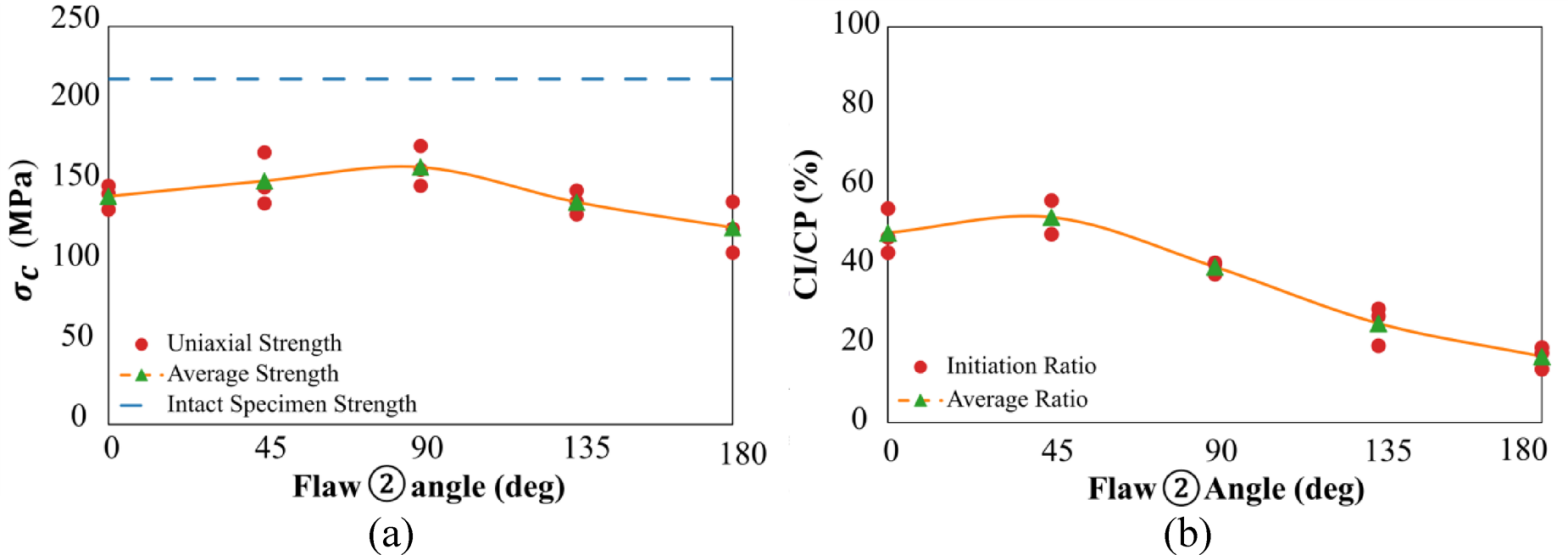
Effect of changing the flaw ② Angle on a: maximum Specimen Strength and b: Crack Initiation Stress Ratio.

This section examines the impact of varying flaw geometries on the strength parameters of specimens. Figure 20a illustrates the maximum observed strength for different flaw geometries and also for intact specimens. As seen in Fig. 20a, the presence of flaws in the rock, on average, led to a reduction in specimen strength. This decrease ranged from 29% (S90 geometry) to 43% (S180 geometry) compared to the intact specimen, which had a strength of 217 MPa.

Among the different geometries, S90 exhibited the highest strength, while S180 showed the lowest. As the flaw angle changed from 0 to 90° (in cases where flaws did not overlap), the specimen strength, on average, increased from 143 to 161 MPa (with the highest value observed in the S90 specimen). In contrast, for S135 and S180 geometries, the strength showed a decreasing trend, reaching its lowest point of 123 MPa in the S180 geometry. It appears that the absence of overlap in S0 and S45 geometries explains their higher strength compared to the overlapping S135 and S180 specimens. Furthermore, the highest strength observed in the S90 specimen indicates less involvement of flaw ② in the crack growth process, which contributed to the specimen’s higher resistance.

Figure 20b shows the crack initiation stress ratio (CI/CP) for different flaw geometries, calculated as the initial crack stress divided by the specimen’s ultimate strength. A higher ratio (closer to 100%) indicates a smaller stress gap between crack initiation and failure, while a lower ratio suggests a larger gap. Non-overlapping geometries generally exhibit higher ratios than overlapping ones. Among them, S45 specimens had the highest ratio (51%), followed by S0 (47%), while S90, S135, and S180 showed progressively lower values (39%, 25%, and 16%, respectively).

The elevated CI/CP in S45 may not only stem from its non-overlapping configuration but also from its crack alignment with the maximum shear stress. Granite’s high strength and this alignment delay crack initiation until higher stress levels, resulting in a higher ratio. Overall, overlapping geometries exhibit a wider stress interval between initial cracking and ultimate failure compared to non-overlapping ones.

#### Cyclic loading

As previously mentioned, cyclic loading with a frequency of 1 Hz was applied to specimens with different geometries at loading amplitudes of 75%, 80%, and 85%. Table 3 presents the number of loading cycles sustained by each geometry under the different loading amplitudes. As shown, in general, increasing the loading amplitude results in a decrease in the number of cycles the specimens can withstand.

**Table 3:** Fatigue life (in cycles) of each flaw geometry under varying loading amplitudes.

Amplitude (%)	Geometry				
	S0	S45	S90	S135	S180
75	92	5084	22,300	4827	425
80	48	235	336	30	257
85	9	5	50	10	310

The test results demonstrate that the S90 flaw geometry exhibited superior fatigue performance at 75% loading amplitude, enduring 22,300 cycles—the highest fatigue life observed. This exceptional performance can be attributed to two key factors: first, the secondary flaw in this configuration appears to remain inactive in the crack propagation process, thereby reducing stress concentration; second, this particular flaw arrangement may delay crack path coalescence. Notably, this outstanding performance under cyclic loading aligns perfectly with static loading results, where S90 showed the highest strength. In contrast, the S0 geometry displayed the poorest performance at 75% amplitude, withstanding only 92 cycles. These results indicate that the flawed geometry in these configurations promotes rapid stress concentration and subsequent failure.

Increasing the loading amplitude to 80% and 85% resulted in a significant reduction in fatigue life across most geometries. For instance, the S90 specimen, which showed exceptional performance at 75% amplitude, endured only 336 and 50 cycles at higher amplitudes, respectively. Increasing the amplitude resulted in a decrease in fatigue life of approximately 98% compared to the fatigue life shown at 75% amplitude for S45, S90, and S135. In contrast, S0, initially the weakest, showed reductions of 48% and 90%, which were less severe than those observed in other geometries. This decreasing trend follows well-established principles of fatigue behavior in materials.

However, the S180 geometry exhibited anomalous behavior. While it showed the lowest strength under static loading, its fatigue performance under cyclic loading displayed an unexpected trend: initial decrease from 425 cycles (at 75%) to 257 cycles (at 80%), followed by an increase to 310 cycles at 85% amplitude. This peculiar behavior can be explained by a shielding mechanism in which the horizontal flaw (180°) acts as a barrier to crack propagation from the inclined flaw (45°). Under static loading, continuous stress application prevents this protective effect from manifesting, while the intermittent nature of cyclic loading allows the shielding mechanism to become operative. These observations clearly demonstrate that flaw geometry not only determines specimen strength but also critically influences the fatigue-life amplitude sensitivity.

## Conclusion

This study investigates crack propagation behavior in jointed rocks using granite specimens containing two artificial flaws: Flaw ① fixed at 45° and Flaw ② oriented at 0°, 45°, 90°, 135°, or 180° relative to the horizontal. The five geometric configurations (S0, S45, S90, S135, and S180) were subjected to uniaxial monotonic and cyclic loading to examine how flaw geometry and loading type influence crack characteristics (type, path, deviation), development sequence, initiation, coalescence mechanisms, and mechanical properties. DIC was employed for objective analysis, eliminating visual interpretation biases, with results presented herein.

- Cyclic cracks were observed in this study, and their occurrence appeared to be governed by both flaw geometry and cyclic stress amplitude.
- Compared with monotonic loading, cyclic loading altered the sequence of crack initiation and propagation.
- Despite the application of cyclic loading, the final crack coalescence patterns remained similar to those observed under monotonic loading, where overlapping flaws resulted in direct coalescence and non-overlapping flaws led to indirect coalescence.
- Compressive strength, crack initiation stress ratio (CI/CP), and fatigue life were strongly influenced by flaw geometry. Fatigue life generally decreased with increasing cyclic stress amplitude unless geometric shielding between flaws influenced crack propagation (e.g., S180 configuration).
- Relative displacement analysis showed that crack initiation mechanisms were not significantly affected by loading type, while crack propagation mechanisms differed between monotonic and cyclic loading conditions.

## Data Availability

The datasets generated during the current study are available from the corresponding author, Hamidreza Nejati, upon reasonable request (Email: h.nejati@modares.ac.ir).
